# Backward masking implicates cortico-cortical recurrent processes in convex figure context effects and cortico-thalamic recurrent processes in resolving figure-ground ambiguity

**DOI:** 10.3389/fpsyg.2023.1243405

**Published:** 2023-09-21

**Authors:** Mary A. Peterson, Elizabeth Salvagio Campbell

**Affiliations:** ^1^Department of Psychology, University of Arizona, Tucson, AZ, United States; ^2^Cognitive Science Program, University of Arizona, Tucson, AZ, United States; ^3^College of Medicine Tucson, University of Arizona, Tucson, AZ, United States

**Keywords:** recurrent processing, figure-ground perception, context effects, ambiguity, thalamus, corticothalamic, cortico-cortical

## Abstract

**Introduction:**

Previous experiments purportedly showed that image-based factors like convexity were sufficient for figure assignment. Recently, however, we found that the probability of perceiving a figure on the convex side of a central border was only slightly higher than chance for two-region displays and increased with the number of display regions; this increase was observed only when the concave regions were homogeneously colored. These convex figure context effects (CEs) revealed that figure assignment in these classic displays entails more than a response to local convexity. A Bayesian observer replicated the convex figure CEs using both a convexity object prior and a new, homogeneous background prior and made the novel prediction that the classic displays in which both the convex and concave regions were homogeneous were ambiguous during perceptual organization.

**Methods:**

Here, we report three experiments investigating the proposed ambiguity and examining how the convex figure CEs unfold over time with an emphasis on whether they entail recurrent processing. Displays were shown for 100 ms followed by pattern masks after ISIs of 0, 50, or 100 ms. The masking conditions were designed to add noise to recurrent processing and therefore to delay the outcome of processes in which they play a role. In Exp. 1, participants viewed two- and eight-region displays with homogeneous convex regions (homo-convex displays; the putatively ambiguous displays). In Exp. 2, participants viewed putatively unambiguous hetero-convex displays. In Exp. 3, displays and masks were presented to different eyes, thereby delaying mask interference in the thalamus for up to 100 ms.

**Results and discussion:**

The results of Exps. 1 and 2 are consistent with the interpretation that recurrent processing is involved in generating the convex figure CEs and resolving the ambiguity of homo-convex displays. The results of Exp. 3 suggested that corticofugal recurrent processing is involved in resolving the ambiguity of homo-convex displays and that cortico-cortical recurrent processes play a role in generating convex figure CEs and these two types of recurrent processes operate in parallel. Our results add to evidence that perceptual organization evolves dynamically and reveal that stimuli that seem unambiguous can be ambiguous during perceptual organization.

## Introduction

**Figure 1:**
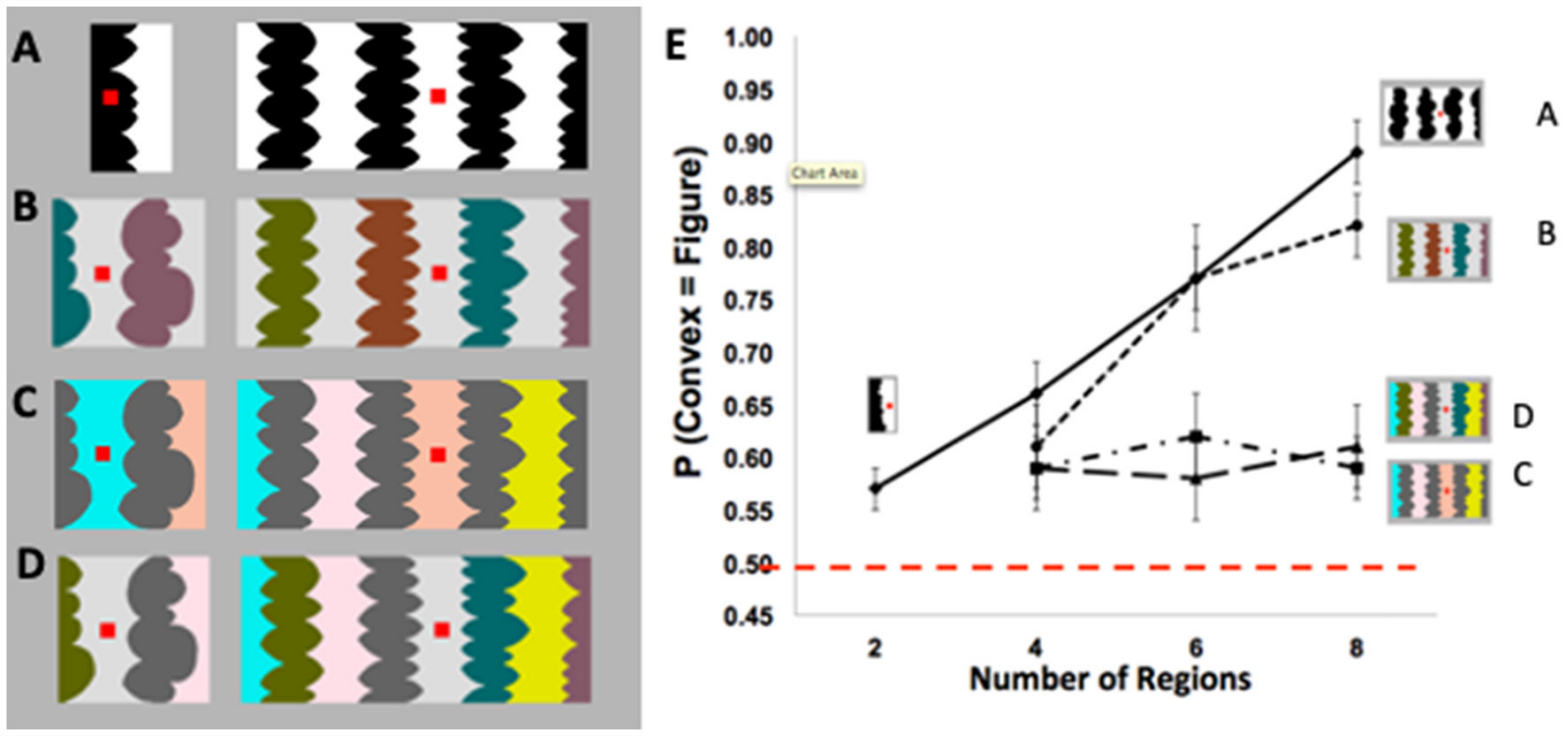
**(A)** Two- (left) and eight-region (right) displays with homogeneous (homo) convex and homo-concave regions. **(B–D)** Four- (left) and eight-region (right) displays comprising **(B)** heterogeneous (hetero) convex and homo-concave regions; **(C)** homo-convex and hetero-concave regions; **(D)** hetero-convex and hetero- concave regions. **(E)** Proportion of convex figure reports as a function of region number for unmasked 100-ms exposures of displays **(A–D)**. P(convex = figure) reports increased with region number only when concave regions were homogeneous (these figures are adapted from Figures 2–5 in Peterson and Salvagio, 2008). Participants' task was to report whether the red probe appeared “on” or “off “the region they perceived as the figure at the nearest border. The dashed red line indicates chance performance (50% convex figure reports). Error bars represent standard error of the mean.

A central function of perception is segregating the visual field into foreground objects and their local backgrounds, yet the underlying mechanisms are not fully understood. Foreground-background perception (i.e., figure-ground perception) was long thought to result from low-level processes in a feedforward perceptual system. Evidence for this view was provided by demonstrations that figure assignment was determined by image-based cues such as convexity. For example, for stimuli like the one on the right in Figure 1A, a large majority of perceivers reported that the convex regions were the figures (e.g., Rubin, 1958; Hochberg, 1971; Pomerantz and Kubovy, 1986). Indeed, convexity was considered the principal figural prior (or “cue,” Kanizsa and Gerbino, 1976). However, using displays exposed for 100 ms, Peterson and Salvagio (2008) found that the probability of perceiving convex regions as figures was only slightly above chance for two-region displays (like Figure 1A, left) and increased systematically with region number to 85–90% for eight-region displays like Figure 1A, right (see Figure 1E). These results indicated that the probability of perceiving convex regions as figures was boosted by global context, a factor that was not previously thought to have an influence. These global context effects (CEs) were observed only when the concave regions were homogeneous (as in Figures 1A, B), but not when they were heterogeneous (as in Figures 1C, D; see Figure 1E).

What processes produce these global CEs? The lack of convex figure CEs for Figures 1C, D ruled out grouping and probability summation, respectively (Peterson and Salvagio, 2008). Goldreich and Peterson (2012) replicated the global convex figure CEs with a Bayesian observer that incorporated a new background prior in addition to the convexity prior. They noted that backgrounds tend (more than figures) to be homogeneously colored. Consistent with this background prior, laboratory research shows that disconnected regions are more likely to be perceived as portions of a single surface when they are homogeneously rather than heterogeneously colored (Yin et al., 1997, 2000). The Bayesian observer also made the novel prediction that the classic eight-region displays like the one on the right in Figure 1A, in which both the convex and concave regions are homogeneously colored, are ambiguous during perceptual organization; ambiguity arises because the background prior of homogeneous color and the object prior of convexity oppose each other for convex regions. This prediction was surprising because the displays do not seem to be ambiguous: a large majority of observers report perceiving convex regions as figure (e.g., Kanizsa and Gerbino, 1976). If this prediction is confirmed, however, that will provide evidence that complex perceptual organization processes take place outside of awareness, even when a single prior was previously considered sufficient. Here, we report three experiments using backward pattern masks to examine the development of convex figure CEs for putatively ambiguous and unambiguous displays like those in Figures 1A, B, respectively, in order to better understand the dynamics of figure-ground segregation.

We are particularly interested in whether feedback from higher to lower levels in the visual hierarchy (i.e., recurrent processes) plays a role in convex figure CEs and in resolving ambiguity during perceptual organization. It is reasonable to assume that the homogeneous background prior entails perceptual completion, which seems to require feedback (Wyatte et al., 2012, 2014; Tang et al., 2014, 2018; for review see Thielen et al., 2019; Kreiman and Serre, 2020). It is known that contextual influences on neural responses are mediated by recurrent processing (e.g., Lamme, 1995; Zipser et al., 1996; Gilbert and Li, 2013). Recurrent processes within the primate cortex modulate the responses of V1 neurons to figures defined by contrasting features inside and outside their receptive fields (e.g., Lamme and Roelfsema, 2000; Lamme et al., 2002; Craft et al., 2007; see Kelly and Grossberg, 2000; Jehee et al., 2007 for models implementing recurrent processes in figure-ground perception). Recently, Self et al. (2019) showed that recurrent input to V1 from a higher cortical level plays a role in resolving a local ambiguity in figure-ground organization. Going beyond cortico-cortical recurrent processing, Sillito and Jones (2002), Jones et al. (2015), and Poltoratski et al. (2019) found that cortico-fugal feedback modulates the neural representation of figures in the primate thalamus. Indeed, cortico-thalamic feedback seems to be automatic; Jones et al. (2015) hypothesized that it iteratively refines local thalamic responses to be consistent with global responses in higher-level cortical areas. Based on this previous research regarding context effects in figure-ground perception, we investigated whether recurrent processing plays a role in convex figure CEs.

**Figure 2:**
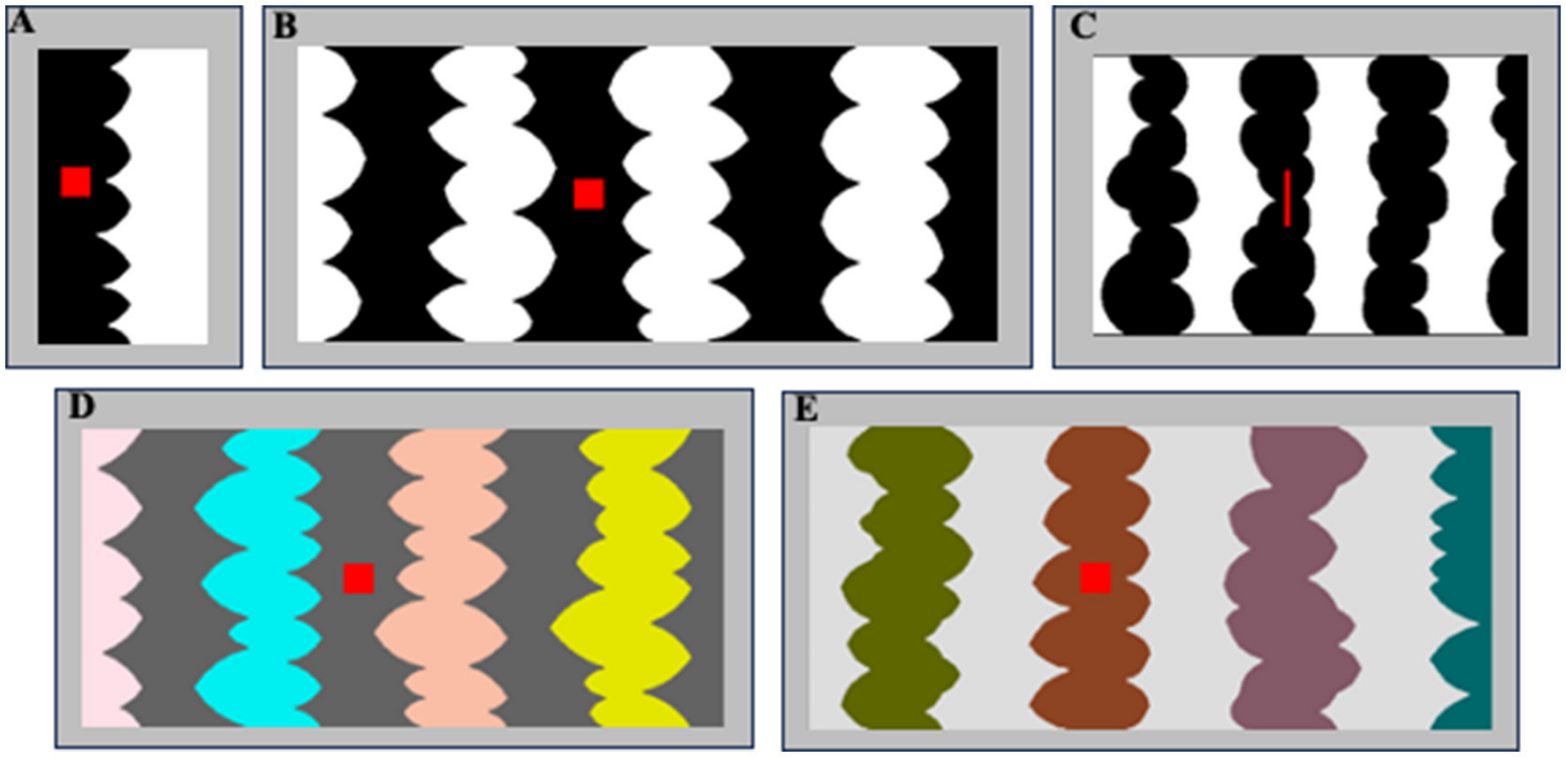
**(A, B)** Sample two- and eight-region homo-convex displays used in Exp. 1A. Convex region(s) are black in **(A)** and white in **(B)**; located to the left of the central border in **(A)** and to the right of the central border in **(B)**. The red probe is on the convex region in **(A)** and off the convex region in **(B)**. **(C)** A sample eight-region display used in Exp. 1B with black convex regions. The red probe is on the convex region to the left of the central border. **(D, E)** Sample eight-region hetero-convex displays used in Exp. 2. Convex regions are HL in **(D)** and LL in **(E)**.

We began by investigating the development of convex figure CEs obtained with the classic displays like those in Figure 1A; see also Figures 2A–C. Convex figure CEs are characterized by substantially higher convex figure reports for eight-region than two-region displays. In the eight-region displays used in all experiments in this article, the concave regions were homogeneous, an essential ingredient for convex figure CEs. In the classic displays used in Exp. 1, the convex regions were also homogeneous. In the displays tested In Exp. 2, the convex regions were heterogeneous. Henceforth, these two types of displays will be labeled *homo-convex* and *hetero-convex* displays, respectively. Test displays were exposed for 100 ms (the duration used by Peterson and Salvagio, 2008) and were followed by a 200-ms pattern mask after interstimulus intervals (ISIs) of 0, 50, or 100 ms. The 100-ms duration during which the test displays were shown is sufficient for feedforward activation through the visual hierarchy (e.g., Lamme and Roelfsema, 2000; Bullier, 2001). Hence, activation from the mask is unlikely to interfere with feedforward activation from the display (e.g., Lamme et al., 2002; Breitmeyer and Ogmen, 2006; Roelfsema, 2006; Di Lollo, 2007; Fahrenfort et al., 2007; Wyatte et al., 2012, 2014; but see Breitmeyer and Ogmen, 2022). However, feedforward activation from a subsequently presented pattern mask can add noise to the substrate for recurrent processing initiated by a preceding stimulus. Perceptual organization that depends on recurrent processes would emerge more slowly as a consequence. Therefore, if recurrent processing is involved in convex figure CEs, the probability of observing CEs should increase with display-to-mask ISI. Moreover, if, as hypothesized, *homo-convex* displays are ambiguous and ambiguity resolution also requires recurrent processes, convex figure CEs may emerge in a longer ISI condition for *homo-* than *hetero-convex* displays. This is because it takes time to resolve ambiguity (Peterson and Lampignano, 2003; Peterson and Enns, 2005; Brooks and Palmer, 2011). The outcome of these experiments will yield insights into the complex interactive processes that lead to the determination of where convex objects lie with respect to borders in scenes.

## Experiment 1

In Exp. 1A, participants viewed two- or eight-region *homo-convex* displays like those in Figures 1A, 2A, B for 100 ms; for each display they reported whether they perceived the convex region as a figure. Displays were followed by a pattern mask at one of three ISIs (0, 50, or 100 ms). Convex figure CEs are defined by significantly higher convex figure reports for eight-region than two-region displays. We found that convex CEs increased in magnitude as the display-to-mask ISI increased from 0 to 100 ms, consistent with predictions if recurrent processing is involved in generating convex figure CEs. In Exp. 1B, we presented narrower eight-region displays (see Figure 2C) in the same display-to-mask ISI conditions used in Exp 1A to investigate whether the recurrent processes implicated in convex figure CEs operate between levels of the visual hierarchy (vertically) or within a level (i.e., horizontally). In both cases, backward pattern masks could add noise to the substrate for recurrent processes. However, within-level recurrent processes take more time as the distance they must travel increases whereas those between levels are substantially less affected by distance (Girard et al., 2001). Therefore, horizontal within-level recurrent processes would be implicated if convex figure CEs emerge at a shorter ISI for narrow displays than for wider displays, whereas vertical between-level recurrent processes would be implicated if convex figure CEs develop along the same time course for narrow and wide displays.

### Participants

Participants in Exp. 1 and all experiments reported in this article were undergraduate students at the University of Arizona who took part to partially fulfill the requirements of an introductory Psychology class. They signed a consent form approved by the University of Arizona IRB before participating. All participants reported normal or corrected-to-normal vision. The data from participants who failed to respond within 3,000 ms on at least 85% of the trials were removed. This standard criterion was applied to all conditions in all experiments. These aspects of the experiments held true for all participants in all experiments reported in this article.

A total of 200 students took part in Exp. 1A; 104 students participated in the follow-up experiment; and 104 students (59 F; 37M) participated in Exp. 1B. The number of participants whose data were removed because they did not meet the standard criterion was eight for Exp. 1A, four for the follow-up experiments to Exp. 1A, and eight for Exp. 1B.

### Stimuli

The stimuli used in Exp. 1A were 112 two- and eight-region *homo-convex* displays (56 per region number condition) comprising alternating low luminance (LL; RGB = 0,0,0) and high luminance (HL; RGB = 255,255,255) convex and concave regions (see Figures 1A, 2A–C). In Exp. 1A, the stimuli were all equal in height (5.65°H) and varied in width (W): Two-region displays were on average 2.92°W (range: 2.45–3.28°; see Figure 2A); eight-region displays were 13.87°W (range: 12.17–15.87°; see Figure 2B). In Exp. 1B, the stimuli were 96 eight-region *homo-convex* displays that were 5.53°H x 8.53°W (see Figure 2C). Regions were deemed convex if their parts, delimited by successive minima of curvature, had positive curvature (cf., Peterson and Salvagio, 2008). Convex regions were LL and concave regions were HL in half of the displays, with achromatic colors reversed in the remaining half. In half the displays, the region to the right of the central border was convex; in the other half, the region to the right of the central border was concave (see Peterson and Salvagio, 2008 for complete stimulus construction details). An invisible rectangular frame around the displays cut the leftmost and rightmost regions of the displays in half, giving the impression that they continued behind the frame. Burrola and Peterson (2014) and Mojica and Peterson (2014) found that without a frame that allows perceptual completion, CEs are not observed.

A red probe was centered vertically on the region to the right or left of the central border. The red probe was a square in Exp. 1A and a narrow bar in Ex. 1B because the individual regions of the narrow displays were necessarily narrower (see Figure 2C). In previous experiments, responses to square and bar probes did not differ (Peterson and Salvagio, 2008).

**Figure 3:**
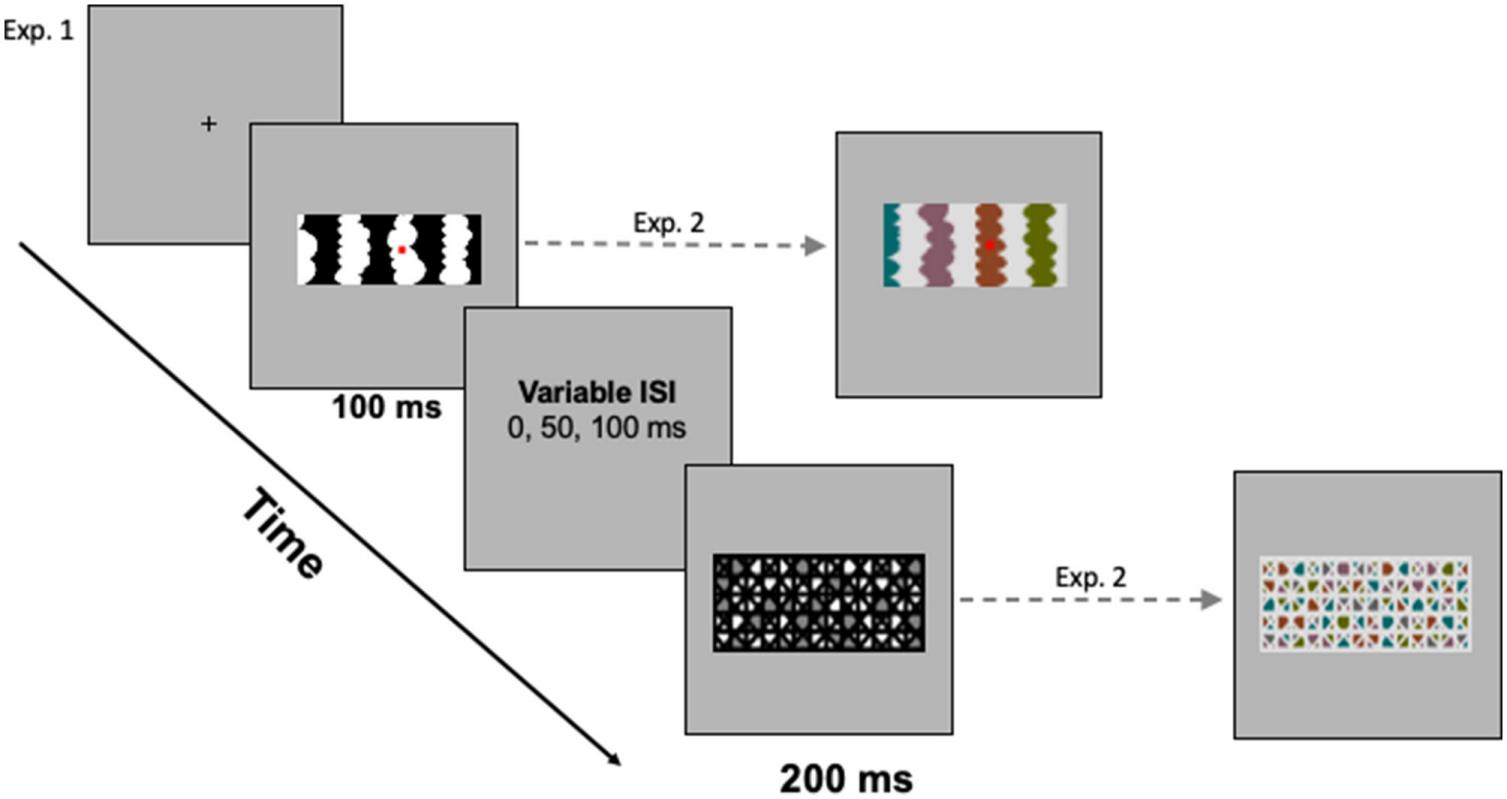
**(Left)** The trial sequence with a sample mask for *homo-convex* displays. **(Right)** Sample stimulus and mask for *hetero-convex* displays. A blank gray screen was shown for a variable interstimulus interval (ISI) between the test display and the mask.

Displays were centered on a medium gray backdrop (RGB = 182, 182, 182; luminance = 11.95 ft-L) that filled the screen (17.7°H x 22.8°W) of a 21-in Sony CRT monitor. The HL and LL regions were equal luminance steps below and above the backdrop; hence, contrast with the backdrop did not serve as a depth cue (see O'Shea et al., 1994). The masks used in Exp. 1 comprised a geometric pattern with white, black, and medium gray regions. In Exp. 1A, the masks were 5.83°H and were 2.98°W for two-region displays and 16.15°W for eight-region displays. In Exp. 1B, the masks were 5.53°H x 9.69°W. A sample mask for *homo-convex* displays is shown in Figure 3.

### Design and procedure

In all experiments in this article, conditions were tested between-subjects to avoid contamination of one condition by another. Participants were assigned via a Latin square to a single region number and ISI condition when they arrived at the laboratory. After signing the consent form, participants were instructed on the nature of figure-ground perception and their task using instructions displayed on the computer; an experimenter read these instructions aloud while they were displayed and stayed in the room during practice trials to answer any questions.

Each trial began with a fixation cross, centered where the central edge of the upcoming test display would be located. Participants were instructed to fixate their eyes on this cross and to press the foot pedal when they were ready to begin each trial. Upon pressing the foot pedal, a single display was presented for 100 ms. The pattern mask (200 ms) was presented 0, 50, or 100 ms after the experimental display. Figure 3 illustrates the trial sequence. The presentation software automatically advanced to the next trial when participants responded or after 3,000 ms had elapsed (a time-out was recorded if participants did not respond within the 3,000-ms window). Viewing distance was constrained by a chinrest mounted 96 cm from the monitor.

On each trial in Exp. 1, a *homo-convex* test display appeared for 100 ms. Participants' task was to report whether the red probe on the display was located “on” or “off” the region they perceived as the figure shaped by the nearest border. This probe on/off task provides a valid and reliable index of figure assignment near a border (e.g., Hoffman and Singh, 1997; Peterson and Salvagio, 2008; Mojica and Peterson, 2014; Peterson et al., 2017). The instructions stated that there were no correct answers in the experiment, that different people see the displays differently, and that the experimenters were interested in participants' first impression of the display. Participants were told that a random pattern would appear after the test display disappeared (this was the mask) and that they only had to look at it this pattern, not respond to it.

On experimental trials in Exp. 1A, each participant viewed 56 randomly presented trial unique *homo-convex* displays in one region number (two- or eight-region) and display-to-mask ISI condition. Participants in Exp. 1B viewed 96 trial-unique *homo-convex* displays. Participants made their on/off judgment regarding the red probe by pressing the top or bottom button on a custom button box. Assignment of buttons to “on”/“off” responses was balanced across subjects. Before the experimental trials, participants completed eight practice trials; none of the displays used in the practice trials appeared in the experimental trials. Participants were left alone to complete the experimental trials.

### Data analysis

The proportion of trials on which the convex region closest to fixation was perceived as the figure/object was calculated for each participant by summing the number of trials on which they reported “on” when the probe appeared on the convex region, and “off” when the probe appeared on the concave region and dividing this sum by the total number of trials on which they responded (i.e., excluding timeouts and responses faster than 200 ms).

### Results

#### Experiment 1A

**Figure 4:**
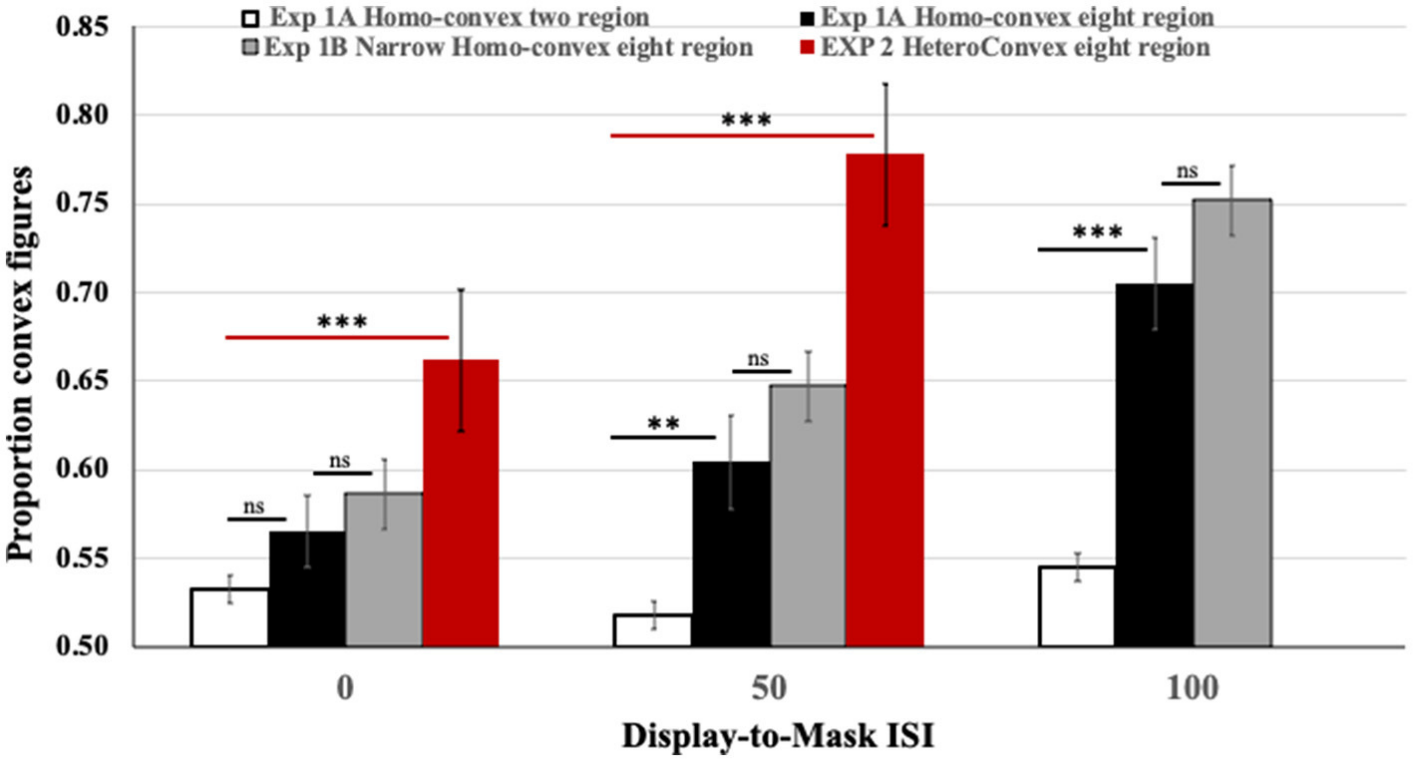
Results of Exps. 1A, 1B, and 2. Black and white: The proportion of convex figure reports for two-region (white) and eight-region (black) homo-convex displays in Exp. 1A. Gray: The proportion of convex figure reports for narrower eight-region homo-convex displays in Exp. 1B. Red: The proportion of convex figure reports for eight-region hetero-convex displays in Exp. 2. Black horizontal lines indicate differences between results for two- and eight-region displays in Exp. 1A. Gray horizontal lines indicate ns differences between results for eight region displays in Exps. 1A and 1B. Red horizontal lines indicate differences between results for two- and eight-region displays in Exp. 2. **p < 0.05; ***p < 0.001; ns, no significant difference. Error bars represent standard errors.

As can be seen in the black and white bars in Figure 4, convex figure reports increased with region number, *F*_(1, 186)_ = 19.39, *p* < 0.001, η^2^ = 0.094 and with display-to-mask ISI, *F*_(2, 186)_ = 4.98, *p* < 0.009; η^2^ = 0.051. Importantly, an interaction between region number and ISI, *F*_(2, 186)_ = 3.06, *p* < 0.05; η^2^ = 0.032, showed that convex figure reports increased with ISI for eight-region displays, *F*_(2, 93)_ = 4.894, *p* =0.01, η^2^ = 0.095, but not for two-region displays, *F* < 1. To represent the magnitude of the convex figure CEs, the difference between convex figure reports for eight- vs. two-region displays was calculated for each ISI condition. This *CE index* was not statistically different from zero in the 0-ms ISI condition [0.033, *F*_(1, 62)_ = 1.629, *p* > 0.20]; it just reached statistical significance in the 50-ms ISI condition [0.086, *F*_(1, 62)_ = 4.12, *p* < 0.05] and was robust in the 100-ms ISI condition [0.16, *F*_(1, 62)_ = 16.43, *p* < 0.001]. The *CE index* was statistically higher in the 100-ms than the 50-ms display-to-mask ISI condition, *p* < 0.002 (see Table 1).

**Table 1:** The proportion of convex figure reports and CE indices as a function of region number and display-to-mask ISI in Exp. 1A and the follow-up Experiment.

	**Display-to-mask ISI (ms)**				
	**0**	**50**	**100**	**200**	**300**
	**Proportion convex figures**				
8-region	0.57	0.60	0.71	0.64	0.71
2-region	0.53	0.52	0.55	0.49	0.53
	**CE index**				
	0.03	0.09^*^	0.16^***^	0.15^***^	0.18^***^

The CE Index is the difference between convex figure reports for eight- and two-region displays.

CE, Context Effect.

^*^p < 0.05; ^***^p < 0.01.

#### Follow-up experiment

To investigate whether convex figure CEs continue to develop longer than 100-ms after the offset of the test stimulus, we presented different groups of participants two- and eight-region displays in 200-ms and 300-ms display-to-mask ISI conditions. We compared convex figure responses in these new conditions to those reported in the 100-ms ISI condition of Exp. 1A in a 2 (region number) X 3 (ISI) ANOVA. A significant main effect of region number was observed, *F*_(1, 159)_ = 33.219, *p* < 0.001, η^2^ = 0.0174, but there was no effect of ISI, *F*_(2, 159)_ = 2.002, *p* > 0.13 (see Table 1).

Together with the follow-up experiments, the results of Exp 1 show that convex figure CEs reached asymptote for 100-ms displays in the 100-ms display-to-mask ISI condition−200 ms after stimulus onset. It is plausible that pattern masks shown 0 and 50 ms after the offset of a 100-ms stimulus (and maybe longer up to 100 ms) interfere with recurrent processing following the initial analysis of the test display, thereby preventing the emergence of convex figure CEs. We continue to investigate the feasibility of this interpretation in subsequent experiments reported in this article. At this point, an explanation holding that the test display-off signal is critical for convex figure CEs and that masks interfere with that signal in the 0-ms display-to-mask ISI condition remains possible (Macknik and Martinez-Conde, 2007); it is shown to be infeasible by the results of Exp. 2.

#### Experiment 1B

To better characterize recurrent processes implicated by the results of Exp. 1A, we compared convex figure reports obtained for narrow eight-region displays in the three display-to-mask ISI conditions to those obtained for the wider eight-region displays in Exp. 1A. The gray bars in Figure 4 show the results. A 2(display width) X 3(ISI) ANOVA showed a main effect of ISI: Convex figure reports increased as display-to-mask ISI increased, *F*_(2, 186)_ = 13.354, *p* < 0.001, η^2^ = 0.125. Neither a main effect of display width, *F*_(1, 186)_ = 2.197, *p* = 0.140, η^2^ = 0.01, nor an interaction between display width and display-to-mask ISI was observed, *F* < 1.0. The finding that convex figure CEs for narrow and wide displays showed the same developmental trajectory over variations in display-to-mask ISI characterizes the recurrent processes as operating between levels of the visual hierarchy rather than within a level (i.e., vertically rather than horizontally).

## Experiment 2

In Exp. 1, we found statistically significant convex figure CEs for *homo-convex* displays in the 50-ms display-to-mask ISI condition and larger convex figure CEs in the 100-ms ISI condition. That masks shown up to 100 ms after stimulus offset interfered with the generation of convex figure CEs is consistent with the hypothesis that recurrent processes play a role. Recall, however, that it has been proposed that *homo-convex* displays are ambiguous because when convex regions are homogeneous the convexity object prior and the homogeneous background prior oppose each other. If this proposal is correct, Exp. 1 may have assessed the need for recurrent processing in ambiguity resolution as well as in convex figure CEs.

In Exp. 2, we used the same procedure with *hetero-convex* displays. *Hetero-convex* displays are unambiguous because disconnected heterogeneous convex regions are unlikely to be completed into a single surface (especially given that they change color only when out of sight in *hetero-convex* displays; Yin et al., 1997, 2000; Goldreich and Peterson, 2012). Only the homogeneous concave regions of *hetero-convex* displays would support perceptual completion into a background. The colored interior and the borders of heterogeneously colored convex regions are likely to be combined when convex figures are perceived (cf., Grossberg and Mingolla, 1985; Kellman and Shipley, 1991; Zhou et al., 2000) but this combination is insufficient for convex figure CEs; homogeneously colored concave regions are necessary (see Figure 1). Exp. 2 will provide evidence regarding whether convex figure CEs *per se* grow with display-to-mask ISI. In addition, a finding that convex figure CEs emerge and reach asymptote in a shorter display-to-mask ISI condition in Exp. 2 for *hetero-* than *homo-convex* displays in Exp. 1 will support the hypothesis that *homo-convex* displays are ambiguous. If that result is obtained, the difference between the ISIs in which equivalent convex figure reports are obtained in the two conditions may estimate how much time is required to resolve the ambiguity of *homo-convex* displays.

### Participants

A total of 67 participants (52F; 15M) were tested in Exp. 2. When they entered the laboratory, they were assigned via an ABBA order to one of two display-to-mask ISI conditions: 0 or 50 ms. The data from two participants were excluded from the analysis because they did not meet our response rate criterion. The data from one additional participant were excluded because they pressed the response button immediately after pressing the foot pedal. A total of 42 participants (28 F) took part in a follow-up experiment in which displays were exposed for 80 ms and were followed immediately by a 200-ms mask. They were assigned via an ABBA procedure to view either the same eight-region displays viewed by participants in Exp. 2 or 56 two-region displays used in Experiment 1A.

### Stimuli

The stimuli used in Exp. 2 were 64 eight-region displays from Peterson and Salvagio (2008, Exp. 3): in half the displays *hetero-convex* regions alternated with *homo-concave* regions; these were the experimental stimuli. In the other half, *hetero-convex* regions alternated with *hetero-concave* regions; these were filler stimuli included to reduce tendencies to form a strategy of always reporting either the *homo* or the *hetero* regions as figures (cf. Peterson and Salvagio, 2008). The choice of which 32 of the 64 displays served as filler stimuli was balanced across participants. As per Peterson and Salvagio, responses to the filler stimuli were not analyzed.

The displays were all equal in height (5.65°) and varied in width, subtending a mean visual angle of 13.59° (range: 11.54–15.65°). The convex regions differed in color. The convex region sharing the central border with the concave region was always gray. The other convex regions were filled with one of four colors: yellow, magenta, cyan, or orange. These colors appeared once per display, and across displays appeared on each of the remaining three convex regions equally often. The concave regions were filled with either HL or LL gray. The convex and concave regions differed in contrast polarity: when the concave regions were HL, the convex regions were LL and vice versa. Samples are shown in Figures 2D, E (as in the other experiments, stimuli were shown on a medium gray backdrop).

In the filler displays, the alternating regions were HL or LL and colored gray, yellow, magenta, or cyan. The two central regions were always filled with HL and LL gray; hence, the central regions in the two types of displays were equated. The remaining colors were used to fill the other regions. The same color was never used to fill two consecutive regions; nor was it used in multiple convex (or concave) regions in a single display. Convex regions were HL in half the displays and LL in the rest. The convex and concave regions differed in contrast polarity: when the luminance of the concave regions was high, that of convex regions was low and vice versa. Michelson contrast at the central border = 0.72. Michelson contrasts at the other borders ranged from 0.62 to 0.78.

The mask that followed the figure-ground display consisted of a geometric pattern that measured 6.0° H x 17.7° W (samples are shown in Figures 2, 3). A mask composed of LL gray and HL colored regions followed displays where the concave regions were LL-gray and the convex regions were HL colors and a mask composed of HL gray and LL colored regions followed displays where the concave regions were HL-gray and the convex regions were LL colors. HL and LL masks followed the filler displays equally often.

### Procedure

In Exp. 2, the trial structure was the same as in Experiment 1 (see Figure 3). Test displays were exposed for 100 ms and followed by a 200-ms mask after an ISI of 0 or 50 ms. The filler displays were randomly intermixed with the *hetero-convex* displays. In other respects, the apparatus and procedure of Exp. 2 were the same as that of Exp. 1. In the follow-up experiment, two- and eight-region displays were exposed for 80 ms and followed immediately by a 200-ms mask.

### Results and discussion

The results obtained with eight-region *hetero-convex* displays in Exp. 2 are shown in red in Figure 4. To assess how convex figure CEs for *hetero-convex* displays were affected by display-to-mask ISI, convex figure reports obtained for eight-region *hetero-convex* displays in Exp. 2 were first compared to those obtained with two-region displays in Exp. 1 (with only one convex region, two-region displays cannot be classified as either *homo*- or *hetero-convex*). The ANOVA showed a main effect of region number, *F*_(1, 124)_ = 45.838, *P* < 0.001, η^2^ = 0.270 and an interaction between region number and ISI, *F*_(1, 124)_ = 5.077, *p* = 0.026, η^2^ = 0.039. Convex figures reports for eight-region displays increased with display-to-mask ISI (as in Exp. 1), whereas convex figure reports for two-region displays did not. The results of Exp. 2 are consistent with the interpretation that convex figure CEs entail recurrent processes.

Unlike Exp. 1, in Exp. 2, convex figures were perceived significantly more often in eight-region than two-region displays in the 0-ms ISI condition, *F*_(1, 62)_ = 10.738, *p* = 0.002, η^2^ = 0.148 (the CE index of 0.13 was significantly >0, *p* < 0.001). This finding is inconsistent with a claim that the absence of convex figure CEs for homo-convex displays in the 0-ms display-to-mask ISI condition of Exp. 1 can be explained by mask-induced interference with a display offset signal as per Macknik and Martinez-Conde (2007). It also suggests that the processes generating convex figure CEs for hetero-convex displays are underway while the test displays are exposed. Replicating Exp. 1, the difference between convex figure reports for eight- and two-region displays was larger in the 50-ms display-to-mask ISI condition (CE index of 0.25), indicating that convex figure CEs for *hetero-convex* displays continue to develop after display offset. Because masks shown 50 ms after a 100-ms display are highly unlikely to interfere with feedforward processing, these results are consistent with the hypothesis that recurrent processes play a role in generating convex figure CEs.

#### Follow-up to Exp. 2

The finding that convex figure CEs were evident in the 0-ms display to-mask ISI condition raised the question of when CEs fist emerge for *hetero-convex* displays. To address this question, we showed 80-ms two-region displays and eight-region *hetero-convex* displays to different groups of participants in a 0-ms display-to-mask ISI condition. No convex figure CEs were observed: convex figures were perceived on statistically equivalent proportions of trials for two- and eight-region displays: 0.56 and 0.60 [*F*_(1, 36)_ <1; the CE index was 0.04]. Together, the results of Exp. 2 and this follow-up experiment suggest that, when object and background priors are not in opposition for convex regions, the processes that produce convex figure CEs in eight-region displays take more than 80 ms after display onset and continue for up to 150 ms (i.e., 50 ms after the offset of the 100-ms display). These findings accord with previous estimates of how long perceptual completion takes although our displays are different from those examined by previous authors (e.g., Sekuler and Palmer, 1992; Ringach and Shapley, 1996; Guttman et al., 2003).

Why do convex figure CEs emerge earlier in time for *hetero-convex* displays (Exp. 2) than for *homo-convex* displays (Exp. 1)? We have attributed this temporal difference to processes that resolve the ambiguity of *homo-convex* displays. This raises the question of whether ambiguity resolution occurs in parallel with the generation of alternative interpretations for *homo-convex* displays or whether it occurs in a later decision process. It is reasonable to assume that perceptual completion processes generating background interpretations for homogeneous regions are underway while *homo-convex* displays are exposed as well as while *hetero-convex* displays are exposed, yet convex figure CEs are evident in convex-figure responses 50 ms later for *homo-* than for *hetero-convex* displays. We found that the cost of ambiguity resolution is approximately constant with increases in the ISI between eight-region displays and the subsequent backward masks: AN ANOVA comparing convex figure reports for eight region displays in the 0- and 50-ms ISI conditions common to both Exps. 1A and 2 showed a main effect of display type, *F*_(1, 124)_ = 15.681, *p* < 0.001, η^2^ = 0.112 (higher convex figure reports for hetero- than homo-convex displays); and a main effect of ISI, *F*_(1, 124)_ = 5.11, *P* = 0.026, η^2^ = 0.04 (higher convex figure reports in the 50-ms ISI condition than the 0-ms ISI condition). No interaction between display type and ISI was observed, *F*_(1, 124)_ = 1.273. This analysis reveals that the disadvantage for convex figure reports in eight-region *homo-* vs. *hetero-convex* displays is present in the 0-ms condition and remains stable as convex figure CEs develop. This result is consistent with the interpretation that ambiguity resolution processes operate in parallel with the processes generating convex figure CEs. Indeed, evidence for convex figure CEs in *homo-convex* displays lagged behind evidence for convex figure CEs in *hetero-convex* displays by ~50 ms: Convex figure reports for eight-region homo-convex displays in the 50-ms ISI condition where convex figure CEs first emerged in Exp. 2 (mean: 0.60; se: 0.04) were statistically equivalent to convex figure reports for eight-region *hetero-convex* displays in the 0-ms ISI condition where convex figure CEs first emerged in response in Exp. 2 (mean: 0.66; se: 0.04), *p* > 0.29. Similarly, convex figure reports for eight-region *homo-convex* displays in the 100-ms ISI condition of Exp. 1A (mean: 0.71; se: 0.04) were statistically indistinguishable from convex figure reports for eight region *hetero-convex* displays in the 50-ms ISI condition in Exp. 2 (mean: 0.78; se: 0.04), *p* > 0.29. These results suggest that resolving the ambiguity of *homo-convex* displays adds ~50 ms to the time at which CEs are evident in convex figure reports.

## Experiment 3

**Figure 5:**
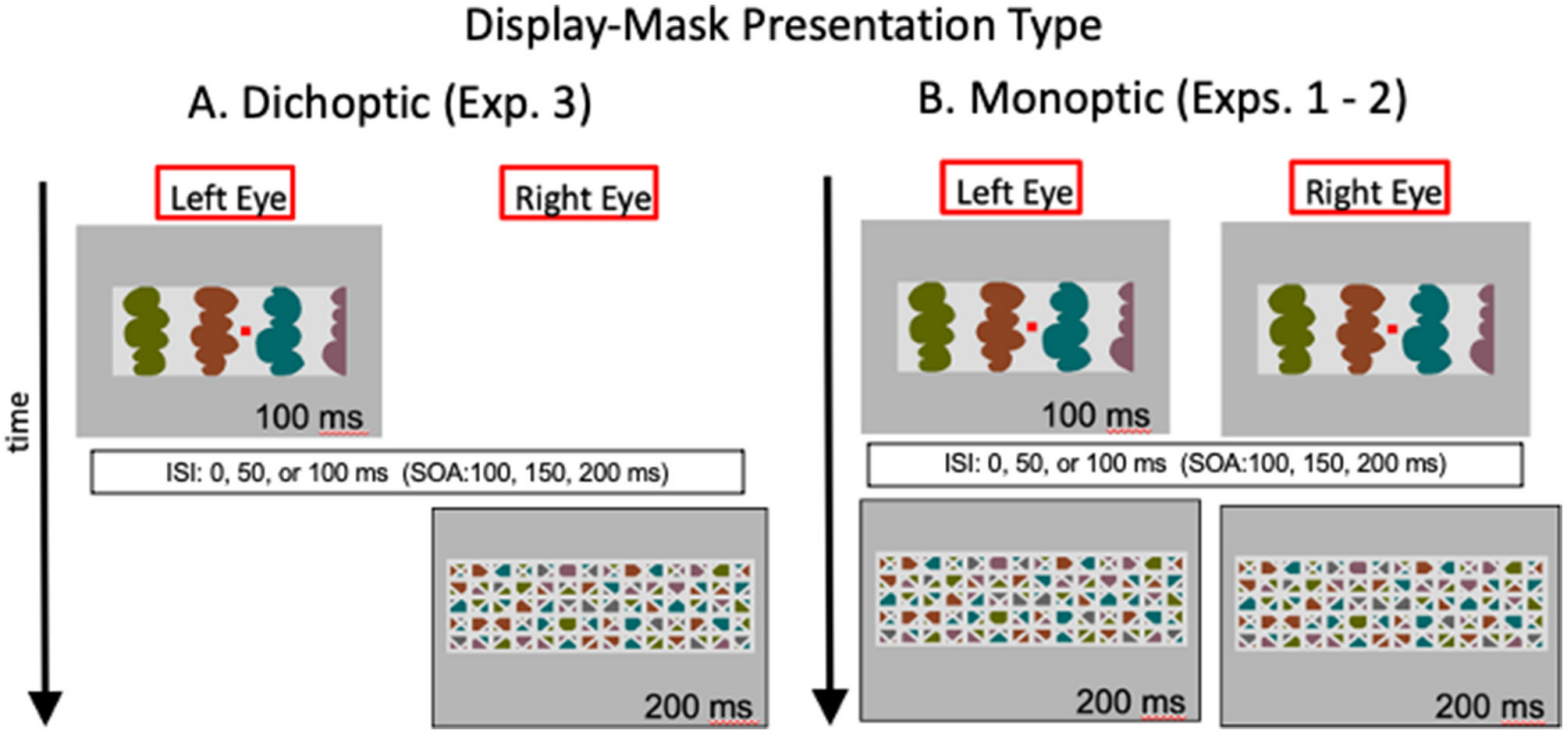
Illustration of the images in the left and right eyes as a function of time with **(A)** dichoptic presentations used in Exp. 3 and **(B)** monoptic presentations used in Exps. 1-2. ISI, interstimulus interval; SOA, stimulus onset asynchrony. *Hetero-convex* displays and masks are illustrated; *homo-convex* displays and masks like the ones in Figure 3 were also used.

We have interpreted the evidence presented so far as consistent with the proposal that convex figure CEs entail recurrent processes. In Exp. 3, we used dichoptic presentations to investigate whether the relevant recurrent processes extend to the thalamus or whether they operate solely within the cortex. In dichoptic presentations, test displays and masks are presented to different eyes, as illustrated in Figure 5A. Thalamic units are monocular. The first units that respond to combined input from both eyes are in cortical area V1. Therefore, with dichoptic presentations, mask-induced activation is absent from the thalamus at least until feedback from area V1 and higher affects thalamic responses. We estimate that time minimally as the time required for area V1 to respond to a stimulus-−40–60 ms after mask onset (Lamme et al., 2002; Tapia and Beck, 2014). Hence, with dichoptic presentations of the display and mask, cortico-thalamic recurrent processing would be free of mask-induced noise until minimally 40–60 ms after mask onset (and perhaps longer if feedback originates in higher-levels than V1). Therefore, if cortico-thalamic recurrent processing plays a role in either or both convex figure CEs and ambiguity resolution, the time course of the convex figure CEs should be shifted 40–60 ms earlier than observed in Exps. 1 and 2 where the test display and the mask that followed it were presented simultaneously to both eyes (as illustrated in Figure 5B). In contrast, if only cortico-cortical recurrent processing is involved, the time course of convex figure CEs and ambiguity resolution should be the same with dichoptic presentations as with the presentation conditions used in Exps. 1 and 2 (the presentation conditions used in Exps. 1 and 2 are referred to as “monoptic presentation” conditions because monocular as well as binocular brain regions respond to the stimuli. With monoptic presentation conditions, mask-induced activation is present as soon as activation begins in the thalamus).

We presented eight-region *homo-* and *hetero-convex* displays and masks to different groups of observers under dichoptic presentation conditions and compared the results to the results obtained for eight-region displays in Exps. 1 and 2, respectively. Only eight-region displays were used because, in previous experiments, convex figure CEs were evident in increased convex figure reports with increases in display-to-mask ISI for eight-region displays but not for two-region displays (see Figure 4 and Table 1). Moreover, the differences between *hetero-* and *homo-convex* displays were evident in convex figure reports for eight-region displays.

### Participants

A total of 215 undergraduate students (147 F; 68 M) from the University of Arizona participated in Exp. 3. Of these subjects, 113 (79 F; 34 M) viewed eight-region *homo-convex* displays and their masks under dichoptic presentation conditions and 102 (68 F; 34 M) participants viewed eight-region *hetero-convex* displays intermixed with filler displays and their masks under dichoptic masking conditions. Data from 23 participants did not meet our response rate criterion; eliminating their data from the analysis left 32 participants in each of the 0, 50, and 100-ms display-to-mask ISI conditions for each display type. Assignment to ISI condition was random.

### Stimuli and apparatus

A haploscope was used with a head and chin rest to present the stimuli dichoptically. In the haploscope, a pair of mirrors reflected to the left and right eyes images that were reflected to them by a second set of mirrors aimed at locations on the left and right sides of a monitor, such that each of these monitor locations were visible to one eye only (see http://www.psy.vanderbilt.edu/faculty/blake/Stereoscope/stereoscope.html). Experimental displays and masks were shown on the left and right monitor locations equally often and, hence, were presented to the left and right eyes equally often.

New sets of eight-region *homo-* and *hetero-convex* displays and filler displays were created in a size visible in the haploscope mirrors (6.45°H X 10.06°W). For each set, masks were created by cropping the masks used in Experiments 1 and 2. The masks measured 7.11°H X 12.42°W.

### Procedure

Participants were seated 51.3 cm from the monitor, with distance controlled by a chinrest. Before the experimental trials, the mirrors of the haploscope were adjusted for each participant individually until the left and right-eye images of a nonius fixation cross were aligned. This procedure assured that images presented on the left and right side of the screen were aligned and centered on the fixation cross.

In each trial the test display and the mask were presented to different eyes. In half of the trials the display was presented to the left eye and the mask to the right eye; in the other half of the trials (randomly intermixed), the display was presented to the right eye and the mask to the left eye. Participants were unaware that the images were presented to different eyes.

Participants who viewed *homo-convex* eight-region displays made their figure reports by pressing one of two vertically aligned buttons on each trial to indicate whether they perceived the black or white regions as figures. For the *hetero-convex* displays participants reported whether an elongated rectangular probe (1.45°H x 0.11°W; RGB = 255, 0, 0; luminance = 4.88 ft-L) centered vertically in either a convex or concave region to the left or right of the central edge appeared to be “on” or “off” the figure (as in Exps. 1 and 2). Peterson and Salvagio (2008) showed that these two types of response produce equivalent results. In other respects, the procedure was the same as in Exps. 1 and 2.

### Data analysis

For *hetero-convex* displays, the data obtained in Exp. 3 were compared to the Exp. 2 data. For *homo-convex* displays, the data obtained in Exp. 3 were compared to the Exp. 1B data because the display widths were similar (the same results were obtained when the Exp. 3 data were compared to the Exp. 1A data).

### Results and discussion

#### Hetero-convex displays

**Figure 6:**
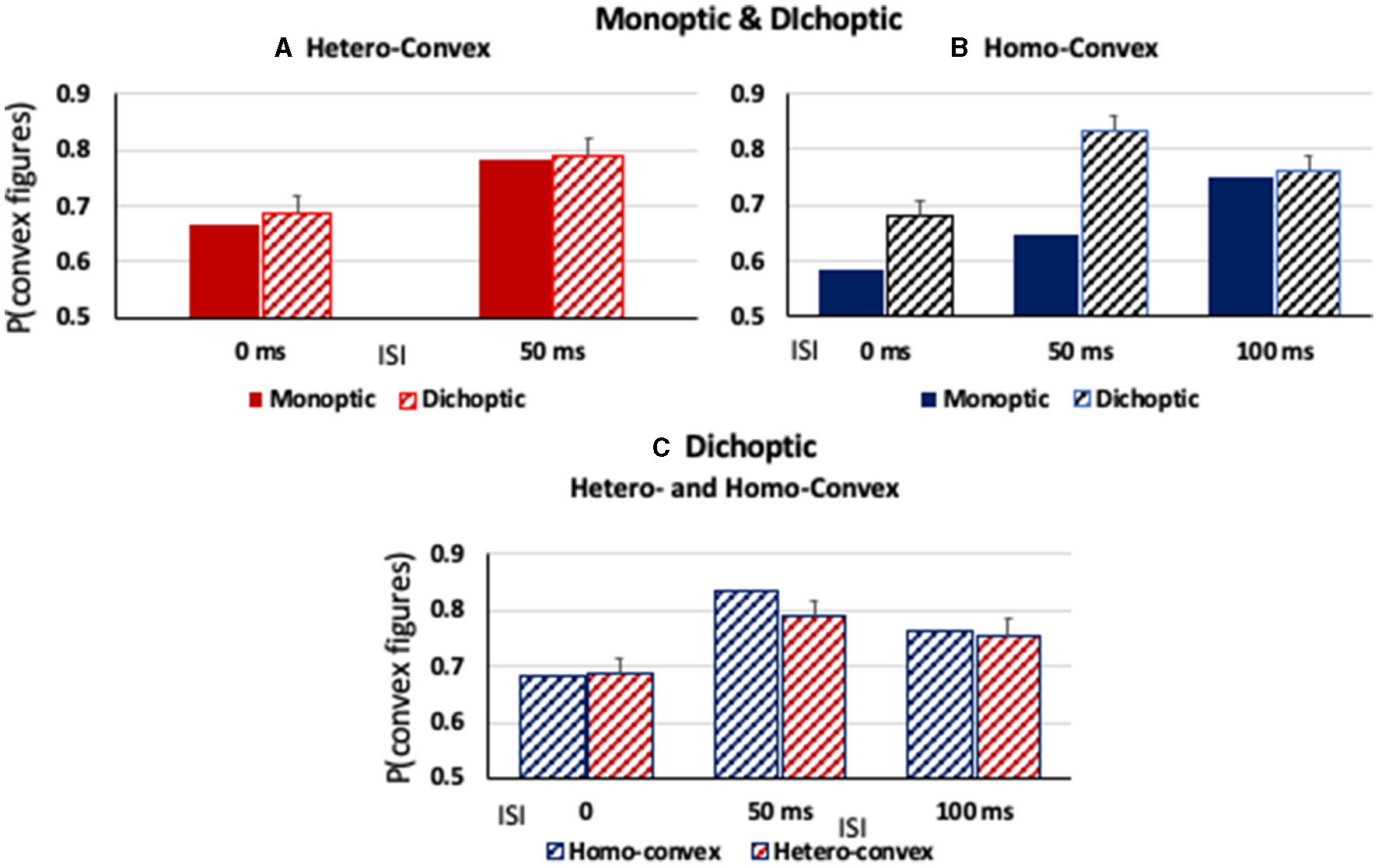
**(A, B)** The proportion of convex figure responses as a function of display-to-mask ISI in monoptic and dichoptic presentation conditions (solid and stiped bars, respectively) for **(A)** hetero-convex displays and **(B)** homo-convex displays. **(C)** The proportion of convex figure responses with dichoptic presentation conditions for homo- and hetero-convex displays (blue and red, respectively). Error bars represent standard errors.

As can be seen in Figure 6A, convex figures were perceived in *hetero-convex* displays equally often in Exps. 2 (monoptic presentations) and 3 (dichoptic presentations) in the 0-ms and 50-ms ISI conditions. A between-experiment ANOVA showed that, for *hetero-convex* displays, convex figure reports increased as ISI increased from 0 to 50 ms, *F*_(1, 124)_ = 10.506, *p* < 0.002, η^2^ = 0.078, replicating Exp. 2 (the 100-ms display-to-mask ISI condition is not included in this ANOVA because it was tested for *hetero-convex* displays only in Exp. 3; it is discussed below). Neither a main effect of presentation type nor an interaction between presentation type and ISI was observed for *hetero-convex* displays, *F*s <1. The absence of an effect of presentation type is consistent with the interpretation that cortico-cortical recurrent processes are involved in generating the convex figure reports in eight-region *hetero-convex* displays. This interpretation is not surprising inasmuch as evidence suggests that convexity is represented in cortical area V4 (Pasupathy and Connor, 1999) and that perceptual completion, a plausible mechanism linking disconnected homogeneous concave regions into a single surface, is represented in the cortex (Kourtzi and Kanwisher, 2001; Rauschenberger et al., 2006; Tang et al., 2014, 2018; Thielen et al., 2019).

#### Homo-convex displays

As can be seen in Figure 6B, convex figure reports for *homo-convex* displays were higher in Exp. 3 (dichoptic presentations) than in Exp. 1B (monoptic presentations) in the 0- and 50-ms display-to-mask ISI conditions, but not in the 100-ms ISI condition where previous experiments indicated convex figure CEs for masked *homo-convex* displays had reached asymptote. This pattern was shown to be statistically significant by main effects of presentation type (Exp) and ISI, *F*_(1, 124)_ = 39.86, *p* < 0.001, η^2^ = 0.24 and *F*_(1, 124)_ = 12.23, *p* < 0.002, η^2^ = 0.09, respectively. An interaction between presentation type and ISI was also observed, *F*_(1, 124)_ = 4.37, *p* < 0.04, η^2^ = 0.034: Convex figure reports for *homo-convex* displays were statistically higher with dichoptic presentations than with monoptic presentations in both the 0-ms and 50-ms ISI conditions (*ps* < 0.008) but not the 100-ms ISI condition*, p* > 0.06, where previous evidence suggested that convex figure CEs for 100-ms displays reached asymptote. This finding suggests that cortico-thalamic feedback occurring up to 50 ms after stimulus offset plays a role in resolving the ambiguity of *homo-convex* displays. When interference from the mask in subcortical areas was removed for a period of time by presenting the mask to a different eye than the experimental display in Exp. 3, ambiguity resolution proceeded without interference and convex CEs reached asymptote in the 50-ms ISI condition, 50 ms earlier than when monoptic presentations of the display and mask were used in Exp. 1.

We next compared convex figure reports for *homo-* and *hetero-convex* displays obtained with dichoptic presentation conditions in Exp. 3 and found that they were equivalent (see Figure 6C). A 2 X 3 ANOVA with the factors of Display Type (*homo*- vs. *hetero-convex*) and ISI (0, 50, and 100 ms) revealed a main effect of ISI, *F*_(2, 186)_ = 10.03, *p* < 0.001, η^2^ = 0.097 but not a main effect of Display Type, *F*_(1, 186)_ = 0.497, *p* = 0.482, nor an interaction between Display Type and ISI, *F*_(2, 186)_ = 0.434, *p* = 0.648. The finding that with dichoptic presentations the convex figure CEs emerge and reach asymptote for *homo-* and *hetero-convex* displays in the same display-to-mask ISI conditions suggests that cortical-thalamic recurrent processes involved in ambiguity resolution occur in parallel with cortico-cortical recurrent processes producing convex figure CEs. If ambiguity resolution occurred later, convex figure reports would reach asymptote in a longer ISI condition for *homo-* than *hetero-convex* displays even under dichoptic presentation conditions.

## Discussion

In Exp. 3 when the test display and backward mask were presented to different eyes, thereby eliminating mask-induced noise in thalamic areas for some time, convex figure CEs emerged at the same display-to-mask ISI for *homo-* and *hetero-convex* displays. This finding contrasts with what was found with monoptic presentations of the experimental display and its mask (i.e., in Exps. 1 and 2), where convex figure CEs emerged later in time for *homo-convex* than for *hetero-convex* displays. We attributed the additional time to ambiguity resolution, suggested by Goldreich and Peterson's (2012) Bayesian observer (cf. Lass et al., 2017 for evidence consistent with this claim from tests of older participants). The results of Exp. 3 imply that ambiguity resolution involves a cortico-thalamic circuit. Moreover, our results suggest that, although feedback to the thalamus may occur even when displays are unambiguous (as in Jones et al., 2015; Poltoratski et al., 2019), it plays an essential role when ambiguity resolution is required.

Further research is necessary to determine how the ambiguity of *homo-convex* displays is resolved. One possibility is that feedback from the cortex enhances local convexity responses in the thalamus, thereby iteratively facilitating their transmission to higher cortical levels (cf., Jones et al., 2015; Poltoratski et al., 2019). Another possibility is that enhanced local convexity responses could bias lower-level border ownership cells (Von der Heydt, 2015) toward the convex side. Note that bias toward convexity is insufficient for convex figure CEs, however, homogeneous concave regions are necessary and evidence for convex figures increases with the number of alternating convex and homogenous concave regions (see Figure 1E). Hence, eight-region *homo-convex* displays are globally ambiguous; this ambiguity is most likely represented in regions of the cortex with receptive fields large enough to encompass eight-region displays (10–14° in the experiments presented here). Thus, it is likely that high levels of the visual hierarchy are engaged in the iterative cortico-thalamic activity that plays a role in resolving the ambiguity of *homo-convex* displays. Indeed, Sillito and Jones (2002) proposed that corticofugal feedback optimizes the thalamic contribution to global integration and segmentation.

Another possibility is that iterative cortico-thalamic activity interacts with cortical mechanisms involved in inhibitory competition between the two possible interpretations of *homo-convex* displays. Lass et al. (2017) found that older participants showed reduced or no convex figure CEs for *homo-convex* displays whereas they showed intact convex figure CEs for *hetero-convex* displays. They attributed their results to impaired suppressive mechanisms involved in inhibitory competition in older participants (cf., Betts et al., 2005, 2009; Anderson et al., 2016). There is some evidence that cortico-thalamic recurrent processing may be slower in older individuals (Walsh, 1976; Kline and Birren, 2007). Given that possibility, aging effects may instead or in addition reveal deficits in iterative cortico-thalamic processing. Using dichoptic presentations with older individuals could be informative in this regard.

Evidence indicates that the pulvinar of the thalamus is involved in attentional selection that requires distractor filtering (e.g., Snow et al., 2009; Strumpf et al., 2013). Like distractor filtering, ambiguity resolution entails a form of selection. Selecting one interpretation of an ambiguous stimulus may occur via fine-tuning cortical responses for that interpretation in one or many levels of the visual hierarchy. Ketteler et al. (2014) made a similar proposal regarding the role of cortico-thalamic recurrent processing in resolving linguistic ambiguity (cf., Mestres-Missé et al., 2017). More research is needed to determine the nature of the mechanisms initiated by cortico-thalamic feedback. Visualizing thalamic responses with high resolution fMRI is one avenue we hope to pursue in this regard. Examining perceptual organization in individuals with thalamic lesions is another.

### Ambiguity resolution during perceptual organization can yield a non-reversible percept

There is no indication that *homo-convex* displays are reversible once the conflict between the object prior and the background prior for convex regions has been resolved and the best fitting interpretation has been found. Ample previous research has shown that, given enough time without mask interference, convex figures are perceived by the vast majority of participants who view *homo-convex* displays (e.g., Koffka, 1935; Rubin, 1958; Kanizsa and Gerbino, 1976; Peterson et al., 1998; Bertamini and Lawson, 2008; Peterson and Salvagio, 2008; Barense et al., 2012; Bertamini and Wagemans, 2013; Spanò et al., 2016). Nevertheless, as suggested by our evidence, multiple interpretations are generated during perceptual organization and the best-fitting interpretation is perceived. This is exactly what is expected in a Bayesian brain – the generation of multiple interpretations for perceptual input before the best interpretation is perceived. Our results show that, for *homo-convex* displays, the processes involved in assigning figure and ground are more dynamic than assumed in traditional theories.

Symmetric figure CEs have also been reported (Mojica and Peterson, 2014). Like convexity, symmetry is an object prior, although since it requires a comparison of the two sides of a region it is necessarily more global than convexity. It also may be a weaker object prior than convexity (Kanizsa and Gerbino, 1976; Pomerantz and Kubovy, 1986; but see Mojica and Peterson, 2014). It would be interesting to examine the time course of symmetric figure CEs to investigate how symmetry interacts with the background prior and how conflict between the two priors is resolved in *homo-symmetric* displays.

### Alternative interpretations

Can the results of our experiments be due to the disruption of feedforward activity in high levels of the visual hierarchy rather than to the disruption of recurrent processes? We consider that unlikely for the following reasons: First, the earlier emergence of convex figure CEs for *hetero-* than *homo-convex* displays cannot be explained by earlier high-level processing of the former than the latter. The *hetero-convex* displays are lower in contrast that the homo-convex displays. Feedforward spikes from low contrast images are delayed relative to those from higher contrast images (VanRullen and Thorpe, 2001, 2002; Wyatte et al., 2012, 2014). Therefore, based on estimates of the time for feedforward spikes to reach the cortex alone, one would expect CEs to emerge earlier in time for *homo-convex* displays than for *hetero-convex* displays. This is the opposite of what we found. Second, that convex figure CEs were no longer delayed for *homo-* relative to *hetero-convex* displays with dichoptic presentations implicates the thalamus in resolving the ambiguity of *homo-convex* displays (although the alternative interpretations may be generated in high levels, ambiguity resolution seems to require thalamic involvement). Third, as mentioned previously, differential difficulty of figure-ground decisions made in high levels cannot account for the differences between *hetero*- and *homo*-convex displays observed in Exps. 1 and 2 because those differences are not evident in Exp. 3.

Can factors other than conflict resolution account for the differences we observed between *homo-* and *hetero-convex* displays? The convex and concave regions in the former displays differ in luminance only, whereas those in the latter displays differ in both color and luminance. There is some evidence that stimuli defined by luminance differences only are processed differently from those defined by both luminance and color differences. For instance, Rivest and Cavanagh (1996) showed that borders are localized better in 2-D space when signaled by two attributes rather than one. But the conflict in our displays doesn't entail differential localization of borders in 2-D space; it involves determining whether the borders are contours of convex or concave objects. Moreover, the finding that the CEs evolve at the same time for *homo-* and *hetero-convex* displays with dichoptic presentations indicates that contour localization differences cannot account for the differences observed with monoptic presentations.

Since all conditions were manipulated between subjects rather than within subjects, might the differences between conditions be attributed to group differences rather than to the manipulated variables? Between-subjects designs were used to eliminate the influence of one condition on another. The difference between two-region and eight-region displays is critical for the CEs. Convex regions are perceived as figures much more often in eight-region than in two-region displays. We were concerned that experience with eight-region displays would contaminate convex figure reports for two-region displays, thereby reducing the difference between those two conditions. Each subject responded to many trial-unique displays within the condition in which they were tested to allow a reliable estimate of behavior in that condition. We do not believe that group differences rather than condition effects account for our results because they are replicated by different groups in different experiments (e.g., Exps. 1A and 1B; Exp. 2 monoptic results were replicated in Exp. 3 dichoptic results; and Exp. 3 hetero- and homo-convex results are not different).

It would be interesting to use a within-subjects design to test the questions addressed here and to include more fine-grained manipulation of ISI. It would be difficult, although not impossible, to present trial-unique displays in a within-subjects experiment, so the conditions would necessarily be somewhat different. Although we did not report the results in the body of the paper, we did test intermediate ISIs of 25 and 75 ms for homo-convex displays with dichoptic presentation conditions and found the results fell between the results obtained for the adjacent ISI conditions.

## Conclusion

The three experiments presented here are consistent with the interpretation that recurrent cortico-thalamic processes are involved in resolving the ambiguity of eight-region *homo-convex* displays and suggest that cortico-cortical recurrent processes play a role in generating convex figure CEs.

## Data availability statement

The raw data supporting the conclusions of this article will be made available by the authors, without undue reservation.

## Ethics statement

The studies involving humans were approved by Human Subjects Protection Program UArizona. The studies were conducted in accordance with the local legislation and institutional requirements. The participants provided their written informed consent to participate in this study.

## Author contributions

MP conceptualized the experiments, performed statistical analyses, engaged in theoretical discussions, and wrote the paper. EC created the stimuli, wrote the programs, conducted the experiments, performed statistical analysis, and engaged in theoretical discussions. All authors contributed to the article and approved the submitted version.
